# *“I want to hear you talk with your heart”*: perspectives on receiving and providing mental wellness supports during the COVID-19 pandemic within a First Nation community in Canada

**DOI:** 10.1186/s12889-025-23723-y

**Published:** 2025-08-14

**Authors:** Laura Jane Brubacher, Bryan Tanner, Ningwakwe George, Sharon Bernards, Melody E. Morton Ninomiya, Ashley Cornect-Benoit, Renee Linklater, Samantha Wells

**Affiliations:** 1 Institute for Mental Health Policy Research, Centre for Addiction and Mental Health, Toronto, ON Canada; 2 School of Public Health Sciences, University of Waterloo, Waterloo, ON Canada; 3 Saugeen District Senior School, Southampton, ON Canada; 4 Health Sciences, Wilfrid Laurier University, Waterloo, ON Canada; 5 Shkaabe Makwa, Centre for Addiction and Mental Health, Toronto, ON Canada; 6 Dalla Lana School of Public Health, University of Toronto, Toronto, ON Canada; 7 Department of Epidemiology and Biostatistics, Western University, London, ON Canada; 8 Department of Psychiatry, University of Toronto, Toronto, ON Canada; 9 250 College Street, Toronto, ON M5T 1R8 Canada

**Keywords:** First Nations, Wellness, Health promotion, Mental health, COVID-19 pandemic, Health and social services, Support, Strength

## Abstract

**Background:**

The COVID-19 pandemic presented unprecedented challenges to local health systems, widening gaps in support and disrupting available care. Within Canada, First Nations communities have been disproportionately affected by the pandemic, which exacerbated an already strained system of appropriate services and supports. As part of a broader community-based participatory research project (*The First Nations Wellness Initiative*), the aim of this research was to explore how the COVID-19 pandemic affected people seeking and providing mental wellness supports within a First Nations community, with an eye to informing ways to enhance community strengths to better address pandemic-related challenges and develop community-identified opportunities for mental wellness promotion.

**Methods:**

From September 2020 to March 2022, one-to-one interviews with people with lived experiences with mental health and/or substance use challenges (*n* = 2) and individuals supporting loved ones with lived experiences (*n* = 7) as well as two focus group discussions (i.e., with community youth (*n* = 5) and frontline service providers (*n* = 5)) were conducted in Saugeen First Nation, Ontario, Canada by a local research coordinator/Knowledge Holder. Individuals shared experiences with mental wellness and/or substance use challenges and experiences accessing/providing mental wellness supports during the pandemic. Recommendations for improving supports during and beyond the pandemic were also provided. These qualitative data were analyzed thematically, using a hybrid inductive-deductive approach.

**Results:**

Challenges faced during the pandemic included difficulties finding and navigating available supports; problems connecting via virtual services; and lack of access to cultural and/or spiritual supports. Participants described relational supports (kinship, friends, the broader community) as well as formal supports (culturally-embedded programs, group supports, youth support group) as key community strengths drawn upon during the pandemic to promote mental wellness. In terms of service provision, challenges balancing differing community needs and concerns were highlighted. Participants shared ideas for expanding and adapting mental wellness promotion and supports to develop a stronger system of care for mental wellness and substance use challenges.

**Conclusions:**

The findings point to opportunities for building on existing community strengths and promoting locally-led, culturally-grounded supports for mental wellness and substance use challenges to enhance capacity of First Nations communities to support their members in the face of public health crises.

**Supplementary Information:**

The online version contains supplementary material available at 10.1186/s12889-025-23723-y.

## Background

Due to the ongoing impacts of colonialism and assimilative policies, pandemic-related challenges to mental wellness promotion have been particularly salient among First Nations communities in Canada. For centuries, First Nations communities have faced discriminatory laws and practices enacted by several levels of government in Canada designed to undermine their independence, with the goal of assimilating First Nations communities into settler society [1–3]. Today, the intergenerational social, cultural, and environmental disruption stemming from these policies underlies a heightened burden of mental health and substance use challenges experienced by many First Nations communities [3–8]. During the COVID-19 pandemic, existing systems of care for First Nations communities, which were already budget-constrained, short staffed, and delivered in a fragmentary fashion [5, 9], faced major additional challenges.

The disruption caused by the COVID-19 pandemic has sparked worldwide discussion around the notion of health systems resilience [10–12]. Resilient health systems are defined by the ability to navigate crises; for example, Kruk and colleagues [13] define health system resilience as “the capacity of health actors, institutions, and populations to prepare for and effectively respond to crises; maintain core functions when a crisis hits; and, informed by lessons learned during the crisis, reorganise if conditions require it” (pg. 1910). This definition involves the capacity of a system to absorb, adapt, and transform in order to continue to provide health services during periods of destabilization and uncertainty [14]. In this regard, health system resilience differs substantively from other forms of resilience, including psychological resilience, which is commonly defined as the ability of an individual (with interplay from familial/community contexts) to positively adapt to the presence of adversity while minimizing negative consequences [15]. This distinction is important, as though psychological resilience has received much attention within Indigenous Health discourse [16–19], we do not consider this framework within the scope of this research. Rather, the goal of this research was to investigate COVID-19-related emergent challenges to local systems of care that are responsible for mental wellness promotion in one First Nations community, to help inform how these supports might be strengthened to respond to future public health crises.

### COVID-19 and mental wellness promotion within First Nations communities

Current evidence suggests that an increased burden of mental health challenges among First Nations communities in Canada existed during the COVID-19 pandemic [20, 21]. This burden may be explained by several factors directly related to the pandemic. For instance, First Nations communities experiencing the acute impacts of mental health or substance use challenges have continued to integrate pathways of healing that are culturally grounded and often emphasize a group setting (e.g., sweat lodge; drumming; sharing circles) [5, 22–24]. However, accessing cultural programs and supports was no longer feasible in many communities due to social gathering limits that were instituted throughout the pandemic, especially during community outbreaks. Research from Ontario, Canada suggests that individuals were less likely to access First Nations cultural supports and activities during the pandemic as compared to pre-pandemic [25]. As was the case in the broader Canadian population, those requiring in-person services for mental health and substance use challenges had their access to care disrupted by social distancing measures instituted to prevent COVID-19 transmission [26]. While there was a shift to virtual care for treatment of these conditions, this shift may have resulted in inequities in access, especially among those experiencing severe symptoms and older individuals who may have difficulties with virtual platforms [27, 28]. Additionally, it has been acknowledged that individuals in need of ambulatory care may have delayed or avoided seeking help altogether due to fear of COVID-19 infection [29]. Outside of care settings, social disruption accompanying the pandemic may have exacerbated mental health challenges for those with existing conditions, while also contributing to a new burden of mental health challenges among those previously less impacted by these challenges [30, 31]. These social and institutional disruptions come at a time of growing severity in outcomes related to the ongoing opioid epidemic, with evidence suggesting that First Nations communities in Ontario have been disproportionately impacted by the rise in overdoses observed during the COVID-19 pandemic [32].

### The present study

To support First Nations-led mental wellness promotion efforts, research is needed to clarify how the COVID-19 pandemic affected– and continues to affect– supports and services for mental wellness within First Nations communities. Drawing on diverse perspectives, including one-to-one interviews with individuals with lived experience of mental wellness and/or substance use challenges or their caregivers; a focus group with a community youth group; and a focus group with frontline service providers, the aim of this research was to investigate mental wellness promotion within the context of the COVID-19 pandemic in one First Nation. We focus on community strengths that support mental wellness, new challenges to systems of care arising due to the pandemic, and opportunities for future mental wellness promotion efforts.

The authors of this article include an Indigenous Knowledge Holder (NG) from Saugeen First Nation (SFN) who, in the role of local research coordinator, conducted all data collection in the community, as well as Indigenous and non-Indigenous researchers outside of SFN who were involved with data analysis, writing, study conceptualization, and administration (RL, SW, SB, LJB, AC-B, MMN, BT). The SFN Community Advisory Circle (CAC), consisting of eight individuals representing the Band Council, youth, and the broader community, provided oversight and guidance on the design and overall conduct of the research. Reporting of this research was guided by the CONSIDER statement for health research involving Indigenous Peoples [33].

## Methods

### Research context: the First Nations Wellness Initiative

This research is part of a broader community-based participatory research project guided by the CAC entitled *The First Nations Wellness Initiative* [34], a collaboration between the Centre for Addiction and Mental Health (CAMH) and Saugeen First Nation (SFN) in southwestern Ontario, Canada. Since 2018, CAMH and SFN have engaged in partnered research focused on mental health and wellness that aims to apply local data to the development and implementation of a wellness strategy that is determined and driven by the community. This work has engaged a variety of research methodologies to centre community members’ lived experiences with mental health and/or substance use challenges, frontline health and social service providers’ perspectives on community supports, and community-identified opportunities to build on strengths [35]. Building on this foundation of partnership, the current study focused on the effects of the COVID-19 pandemic on SFN community members’ experiences of mental wellness and substance use, as well as the availability and appropriateness of mental wellness supports.

### Saugeen First Nation (SFN) and effects of the COVID-19 pandemic

SFN has a population of approximately 2,000 members, 800 of whom live on reserve (SFN Membership Department, March 4, 2022). In July 2021, due to elevated rates of confirmed COVID-19 cases (*n* = 130; 16.9% of members living on reserve) the community declared a State of Emergency. Entire households experienced periods of quarantine and people of all age groups were affected. The community mobilized an array of supports, including a field hospital, with the support of the Canadian Red Cross; food hampers for those in quarantine, with financial and food donations provided by neighbouring communities and the SFN food bank; and financial assistance provided by community leadership to all residents in the case of employment disruptions.

In response to the challenges created by the pandemic, as well as other crises the community experienced during the study period, we adapted our methods to ensure the research and knowledge sharing approaches continued to be feasible, ethical, and respectful, and resulted in outputs that would be most valuable to the community. This included a flexible data gathering timeline, as well as adjusting the location and pacing of interviews as per participants’ needs and preferences.

Research ethics review for this study was provided by the Centre for Addiction and Mental Health Research Ethics Board (REB #: 2018-011). Research protocols were conducted in accordance with the Declaration of Helsinki. The study was also approved by the Community Advisory Circle in SFN. The data that was gathered is owned by SFN.

### Data gathering

From September 2020 to March 2022, qualitative data were gathered through focus groups and interviews with community members about the topic of COVID-19 and community experiences with mental health and substance use and services/supports. Due to social gathering restrictions and lockdowns, data collection occurred over several distinct periods. All participant recruitment and data gathering were conducted by the local research coordinator/Knowledge Holder from SFN (NG), who developed a relational and empathetic approach to engaging with community members. Individuals were recruited to participate by word-of-mouth and by personal invitation. All participants in interviews and focus group discussions provided informed written consent, as well as permission to audio-record.

#### One-to-one interviews

Nine participants shared their experiences in a one-to-one interview: two individuals with lived experiences of mental health and/or substance use challenges (one female; one male), as well as seven individuals supporting loved ones with lived experiences (five females supporting five females; and two females supporting two males). Ages of participants ranged from 23 to 66 years. Participants were asked to share their experiences related to the COVID-19 pandemic and its impacts on mental wellness and/or substance use challenges for themselves, their loved ones, and the community; experiences accessing services and supports during the COVID-19 pandemic; and recommendations for improving services and supports both during and beyond the COVID-19 pandemic [see *Additional File 1* for the full semi-structured interview guide]. Interviews ranged in length from 47 to 118 min (average interview duration = 76 min) and took place between April and October 2021. The research coordinator made repeated attempts to involve more participants in interviews, particularly people with lived and living experience; however, this was exceedingly challenging due to various, compounding traumas individuals were experiencing in the study period. This resulted in several ‘no-shows’ or last minute cancellations. The difficulty connecting with people with lived experience meant that their perspectives had to be considered together with the perspectives of those supporting loved ones during analysis to increase rigour, due to the small sample of individuals with lived experience.

#### Focus group discussions

Two focus group discussions were facilitated: one with members of a recently formed youth group in SFN (*n* = 5 participants; two female, three non-binary, ages ranging from 18 to 23 years) (discussion duration = 59 min); and the other with local health and social service providers (*n* = 5; all female, ages ranging from 40 to 70 years) (discussion duration = 164 min). For both discussion groups, a Traditional Healer from the community was present to provide support, if necessary. Both focus groups were held in October of 2021.

**Fig. 1:**
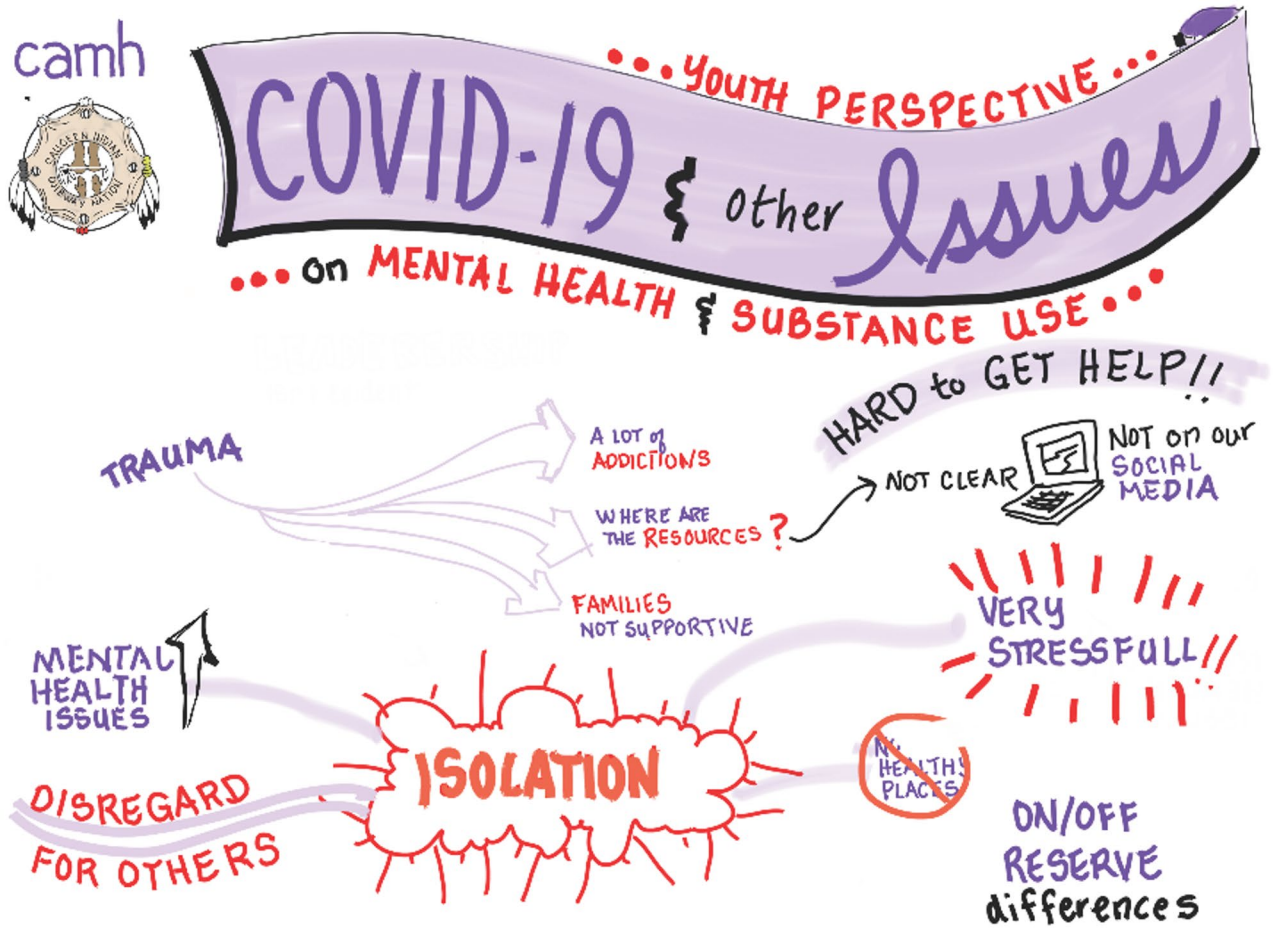
Graphic illustration of youth discussion: Impacts of the COVID-19 pandemic

**Fig. 2:**
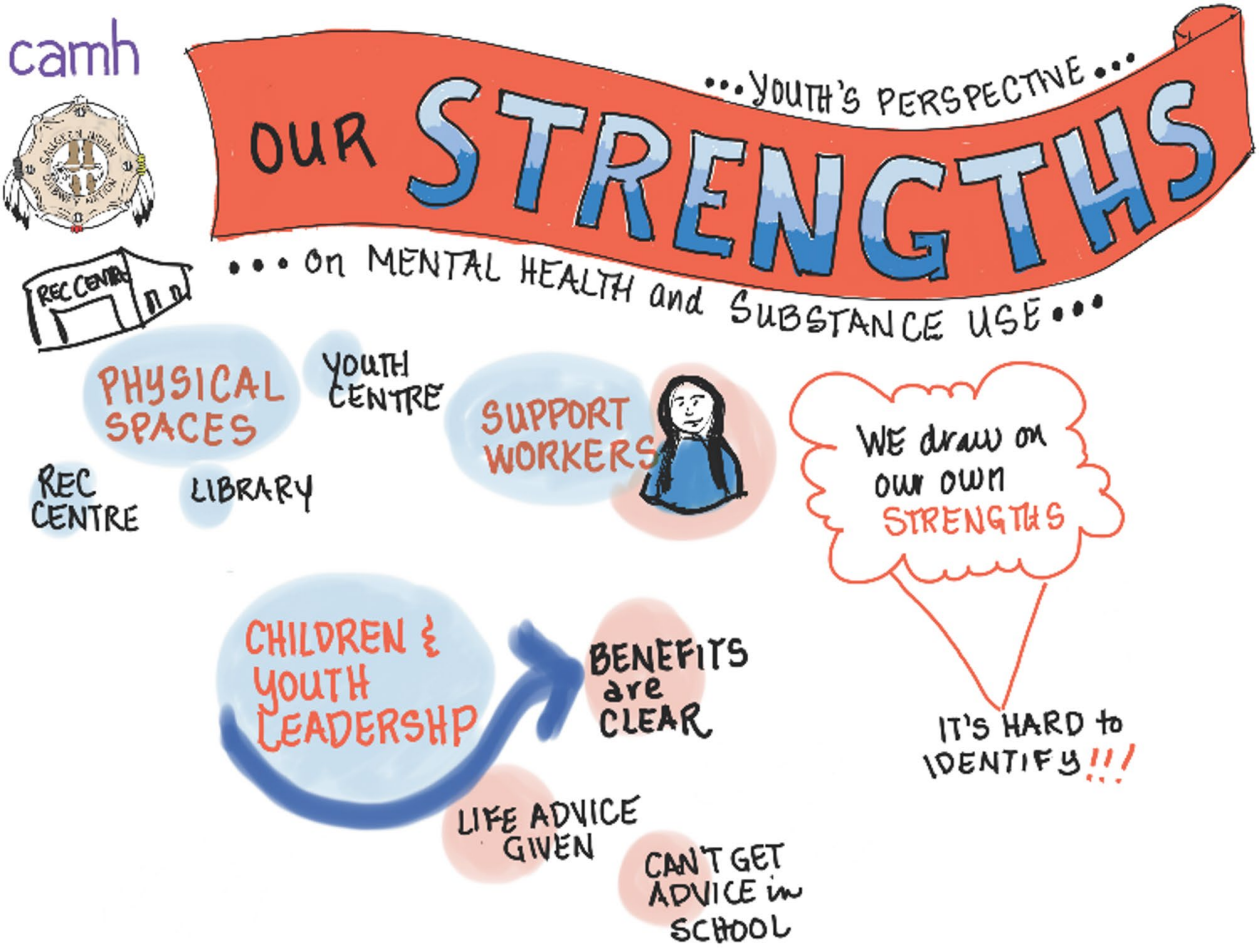
Graphic illustration of youth discussion: Key community strengths identified

**Fig. 3:**
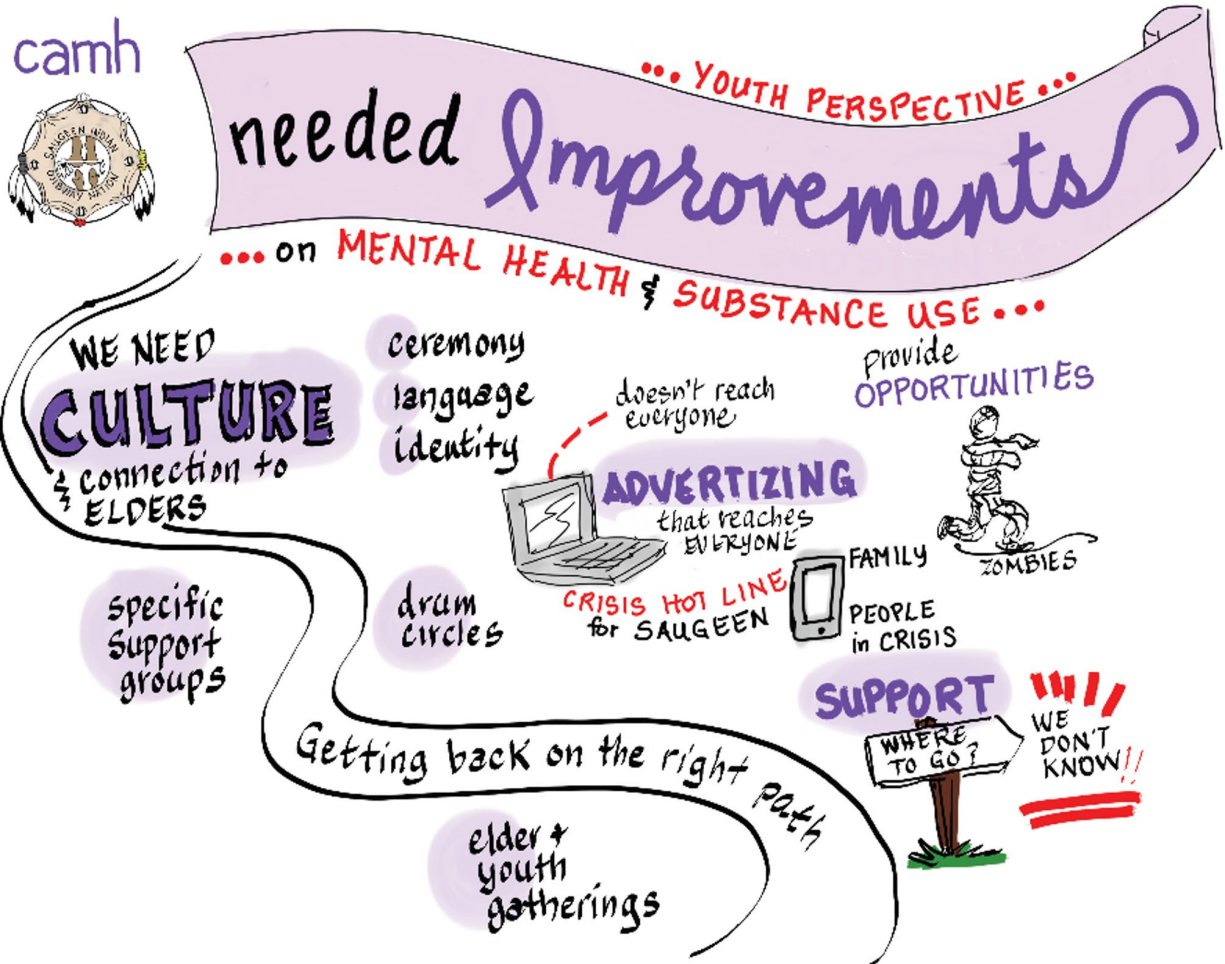
Graphic illustration of youth discussion: Key suggestions for improvements

For the youth focus group, the discussion focused on challenges experienced with mental wellness and/or substance use during the COVID-19 pandemic; strengths in the community; and improvements that can be made to supports in the community. A graphic illustrator also attended to provide a visual reflection of the discussion [Figs. 1, 2 and 3].

The service provider focus group involved discussion of the challenges experienced in providing mental wellness and/or substance use programming or services during the COVID-19 pandemic; supports providers needed to address these challenges for service recipients; as well as potential mental health challenges for providers themselves [see *Additional File 2* for focus group discussion guides].

### Data analysis

Audio-recordings of interviews and focus group discussions were transcribed verbatim. Data analysis used a hybrid deductive-inductive thematic coding approach [36], with the deductive informed by the overarching research questions and objectives, and the corresponding interview guide, and the inductive being data-driven, guided by a grounded theory approach [37]. Codes were iteratively generated, refined, and consolidated into a parsimonious codebook that fit the data [38]. *QSR NVivo© (Release 1.5)* software was used for organization and retrieval of codes and interview excerpts. In addition, memos were used in parallel with the coding process to generate additional insights and engage in reflexivity [39]. Preliminary analyses were conducted by LJB.

Analyses were reviewed by the local research coordinator/Knowledge Holder (NG) who conducted the interviews. As a member of the participating community, NG provided essential feedback and additional community and cultural context to ensure data were interpreted appropriately. This form of debriefing, in addition to the triangulation of data across sources (i.e., participants) and methods (i.e., interviews and focus group discussions) contributed to validity and credibility of the findings [40].

## Results

### Challenges faced during the COVID-19 pandemic

As described in further detail below, all participants and groups identified challenges they experienced during the COVID-19 pandemic. Participants with lived experiences, those supporting loved ones, and youth participants particularly emphasized the challenges of finding and navigating available supports during the pandemic, as well as accessing virtual supports and necessary cultural and/or spiritual supports. Service providers shared their challenges balancing community concerns and their experiences with stress and burnout as they cared for others.

#### Difficulty finding and navigating supports: “you couldn’t just go somewhere to get help”

Across interviews and focus group discussions, participants noted significant barriers to accessing formal services for mental wellness and/or substance use challenges. As noted by one participant, *“you couldn’t just go somewhere to get help.”* Participants said they were unsure of what services were actually available during the pandemic, and had difficulty navigating what was available. For example, one participant with lived experience noted:

Because of like all the COVID precautions and everything, we couldn’t figure out where to go. Like I called a bunch of different places and I was kind of just getting the run-around. They would say, ‘call this place, call this place, call this place’, and so I got frustrated (Lived experience interview participant).

Another participant who was supporting a loved one with lived experience shared: *“[My daughter] tried to reach out and tried to get help for herself as well*,* at one point*,* but because of COVID and everything she kind of just*,* you know*,* was pushed aside…and because it was lockdown*,* there was nobody there to help her.*” Likewise, youth participants shared experiences of isolation, stress, and not knowing what supports were available. In the words of one youth, *“I didn’t know who to reach out to because no one was working at the time…it felt like I was trapped in my house because we [were] in lockdown…it was really stressful.”* In addition to a lack of availability of services amid the peak of the pandemic, a few participants noted challenges accessing needed supports even once services were able to function again in some capacity, pointing to a pandemic-induced ‘backlog’.

#### Challenges with virtual services and supports: “I want to hear you talk with your heart…I can’t hear that talking to a computer”

While some services and supports moved from in-person to online programming (e.g., via ZOOM™ videoconferencing software), there existed recognition among participants that technology ‘worked for some, but not for others’ in terms of providing effective support [Figure 1]. Lack of reliable computer and/or internet access was a cited barrier to accessing virtual supports. One person supporting a loved one noted that older individuals do not always have a computer or may not be familiar with its use, while others encounter internet or phone service issues, especially those living in ‘dead zones’ where internet connections are unreliable at best. One person supporting a loved one shared: *“so she didn’t have the– you know*,* means to do the online groups and stuff and she just kind of got isolated.”*

Beyond access issues, one person with lived experience shared the barriers they faced with ‘opening up’ via virtual supports, thus influencing the effectiveness of the support for them:

Because it was during that COVID shutdown, and it was over webcam, actually, that [the doctor and I] talked. So, it wasn’t really face-to-face, in person…I found it more challenging to sit with someone and tell them about you and how you feel inside, than really being in person, because… It was kind of hard to talk to people in general, about that kind of stuff… Because when you’re in person, you can tell someone’s body movements. In virtual it’s kind of, you can, but it’s different, so I didn’t really feel like I could open up or really be myself. And usually when that happens, I close off, I just shut down kind of, and then I don’t necessarily get everything out (Lived experience interview participant).

Similarly, service providers noted a challenge in trying to connect with clients virtually. As one provider shared, *“I prefer face to face. I want to hear your voice…I want to hear you talk with your heart. I want to hear that. I can’t hear that talking to a computer.”* Another provider stated the importance of personal presence in service provision: *“I personally don’t really want to talk to someone via Zoom™*,* especially an Elder or someone who is suffering. I have to have like their presence*,* so you can feel their energy*,* past energy*,* that kind of stuff.”*

#### Challenges accessing cultural and/or spiritual supports: “it’s a part of who we are”

Participants supporting loved ones as well as one service provider referenced their inability to receive desired cultural and/or spiritual supports during the COVID-19 pandemic, such as a Sacred Fire. This was mentioned by one participant as especially challenging after the loss of a community member. Another shared that a Sacred Fire is “*very integral in our practices. It’s a part of who we are”* and that COVID-19 restrictions preventing the gathering of individuals around a Sacred Fire were upsetting and disrupted the grieving and healing processes in the community:

To be told that something that’s very deep in our ways, something that helps us through our prayers, for healing, whatever it is, that fire has something special. And we’re told from within our own community that we can’t do it (interview participant supporting a loved one with lived experience).

#### Challenges balancing differing community needs and concerns: “damned if you do; damned if you don’t”

Service providers identified challenges in determining how best to offer group supports during and post-pandemic, which had previously been very well-attended. In particular, participants expressed the tensions inherent in balancing concerns around risk of infection with the need for support that is afforded by in-person group programming. For example, while one provider expressed concern over unvaccinated community members putting others at risk of contracting COVID-19, particularly Elders, another perceived that it was divisive to not offer programs to unvaccinated people: *“So it’s conquer and divide*,* right? Conquer and divide against our own people when our ways have always been to gather and that’s how we heal*,* right?”* They went on to say that, *“we work for the community*,* not just for certain ones.”* Similarly, a participant emphasized this challenge of negotiating differing community needs and concerns:

You want to do the programming and you want to be able to have workshops and gatherings…it almost seems as if you’re damned if you do and you’re damned if you don’t. [In reference to a prior event hosted]: We had emails flying at us with regards to ‘oh that’s so unsafe. Who’s following pandemic rules? Is the facilitator fully vaccinated? Have they been tested for? They are probably bringing COVID into the community’, you know all these issues that came up. And it was just like oh my God, these people, they’re professionals. They understand what COVID is and we’re following the protocol…How do you help people when you’ve got other people coming in and putting up road blocks and shutting the door and saying no, you can’t do that (Service provider focus group participant).

#### Stress and burnout among service providers: “so it’s just trying to keep our spirits going”

Service providers spoke to the challenges inherent in their roles, and the importance of ongoing community care and self-care. Two service providers referenced experiencing burnout, using the following analogy *“It’s just like you go to work and it’s like everything’s full blast. And then you finally get away and the volume is down and it’s just a [makes humming noise].”* Another participant responded in agreement, *“A little hum in the back.”*

One participant noted that sometimes they face people in the community who are upset, angry or frustrated, reflecting enduring impacts of the pandemic, which can affect those providing frontline care: *“So it’s just trying to keep our spirits going. The longer this COVID stuff goes on*,* the harder it is on our vulnerable in the community*,* the angrier they get with the staff*,* right?”*

Another service provider described the need to *“take care of ourselves too [as service providers]*,*”* particularly as any crisis affects them not only as service providers, but also as friends or relatives of others in the community who are being impacted in different ways by the pandemic. They also noted that staff debriefing needs to occur more intentionally in situations when staff have responded directly to a crisis situation:

When you respond to a crisis you’re going out by yourself to go see somebody that’s suicidal. What do you do to get out of that state? You take it home and you’ve got people coming in. And there’s a lot of people that are suicidal right now because of the pandemic. So it’s really, really important (Service provider focus group participant).

### Community strengths

Participants in interviews and focus group discussions associated their experiences in the COVID-19 pandemic to strengths within their families and the community at-large, noting that these strengths, that are embedded in community culture and thus have a long history within the community, helped to buffer the impacts of the pandemic or helped them cope. This discussion of strengths was consistent across all participant groups.

#### Relational supports: “we all stand behind one another and support each other”

Most participants spoke to the strength of support rooted in their relationships with kin, friends, and/or the broader community and the importance of this relational support to mental wellness. As one participant shared, during isolation, *“[a friend or family member] would come by and sit at the other side of the lawn and talk to me…it was friends and family that got us through it.”* Others underscored a general ‘sense of community’ and being *“able to pull together in times of crisis and be there to support whatever is going on.”*

However, isolation, gathering restrictions, and other public health and social measures made it difficult for members of the community to draw on these community strengths; for example, as a participant shared, *“as community members*,* we all stand behind one another and support each other in these times and you can’t do that now with COVID.”* This was noted as especially painful for those experiencing grief and loss during the pandemic, such as not being able to attend funerals or gather with others in ceremony.

#### Strengths embedded within the system of formal care supports: “we know who we can call on to help out”

Within the formal system of health and social care for those experiencing mental wellness and/or substance use challenges, service providers in particular underscored community strengths that existed long before the pandemic. For example, service providers noted a system of rapid communication among staff and out to the community (i.e., “*Moccasin telegraph*”, or word of mouth) to provide urgent support when needed; culturally-grounded supports and trainings offered to the community (e.g., programs for learning *“survival skills”)*; and the presence of outreach workers doing home visits. One participant spoke to the consistent and reliable offering of group supports, that *“[a group program] was always going to be there*,* twice a week*,* no matter what.”* Others spoke to the close collaboration among different programs offering services in the community, such as collaborating to distribute resources and provide holistic support (e.g., Food Bank and Ontario Works staff), or through channels of communication across staff as to how to support clients with available programming:

I think us getting together as program managers and staff kind of gives us a good foundation for being able to support not just each other, but the community as a whole. And it’s nice that we can share that information so it’s not just one person scrambling, trying to figure out how we’re going to help this person. We’ve all got, you know, working together, we know who we can call on to help out with those resources (Service provider focus group participant).

Youth participants indicated several community strengths that exist among and for youth that can be drawn upon for support, such as facilities that focus on youth, the availability of support workers, and youth leaders themselves [Figure 2]. They noted their plan to become an official youth ‘council’ to extend support to their peers, as *“that was kind of the main goal when we got together*,* was being a safe place for the youth and then it kind of morphed into us just working on projects…[our] main goal is to be there for youth.”* They have since named themselves the ‘Youth Support Group’.

### Opportunities for the future: Building on strengths to buffer impacts of future compounding health crises

Participants with lived experiences, those supporting loved ones, members of the youth group, and service providers all identified recommendations on how to build on community strengths to buffer the impacts of future crises and foster wellness.

Some of these recommendations pertained to *navigation of existing supports*, recognizing that pre-existent service gaps were made more pronounced during the COVID-19 pandemic, particularly the challenges of determining what support was available and where to access it. A participant noted that some form of liaison or care coordinator would be helpful, such as *“some sort of access– like just somebody to do all that calling around [to supports]”*. Similarly, another suggested creating a system or network of mental wellness and substance use supports, activated by a single phone call to a crisis line (a *“chain reaction”*). Other participants emphasized that communication of available supports was lacking during the pandemic and people were reliant on social media channels which are not accessible to all community members (Elders, especially). These accessibility challenges also extended to wellness programming, as virtual events that rely on computer access and computer literacy were not available to all community members. In light of these challenges, participants identified a need to consider alternative forms of communication (e.g., community radio, hard-copy materials) with broader accessibility to communicate available supports, and to expand these considerations to how health promotion and programming is delivered when in-person support is not possible.

Other participants *characterized the types of supports* needed in order to foster wellness amid future crises. These included wholistic kinship supports, spiritual support, as well as continued prioritization of culturally-embedded supports, such as those oriented around a shared cultural gathering or healing ceremony (e.g., sweat lodges; drumming circles). A need was expressed by people with lived experience for stigma-free support, as well as reduced stigma around mental wellness and substance use challenges, such as dismantling the myth that if *“you’ve [done] drugs*,* you’re automatically a drug addict for life.”* Participants expressed appreciation for group supports that have worked in the past (e.g., gender-specific, or focused on a particular mental wellness or substance use challenge) and recommended enhancing group supports moving forward.

Youth focus group participants made a number of suggestions for ways to improve mental wellness supports specifically for youth, including cultural connections and practices, improved information and awareness of supports that are available, and overall, more activities and opportunities for youth to engage in [Figure 3]. In particular, youth desired more teachings from Elders, and to incorporate ceremony, language, and teachings into youth meetings and events to strengthen cultural identity. Building on existing relational supports in the community, youth recommended more *“Elder and youth gatherings”* or, more broadly, programs that connect younger generations with older youth and other mentors in the community. These were described as opportunities to be surrounded by *“good people”* and receive *“real-life advice”* to promote mental wellness and decrease the risk of substance use challenges. Finally, a concern of the youth was the presence of “zombies”, a name that the youth participants had given to substance users in the community who seemed to be walking through life struggling to find purpose or meaning beyond the use of substances. The youth felt that these individuals would especially benefit from initiatives that centre culture and identity.

## Discussion

The COVID-19 pandemic brought to light critical gaps in systems of healthcare worldwide [10]. Ensuring health systems are resilient to future crises is a key requirement of functional systems of care so that they can continue to nurture a healthy populace even in times of disruption [13]. In this article, we detailed findings from a qualitative research project that explored the impacts of the COVID-19 pandemic on mental wellness promotion efforts in one First Nation community in southwestern Ontario, Canada. Drawing on diverse perspectives of people with lived experiences with mental health or substance use challenges, youth, and community service providers, we highlighted individuals’ experiences and insights of mental wellness and substance use challenges that were exacerbated by the pandemic. In sharing their experiences, participants revealed contemporary challenges arising from the pandemic, key community strengths that provide a foundation for ensuring mental wellness, and finally, hopeful opportunities for future public health initiatives targeting mental wellness promotion within the context of a First Nations community.

A body of research on health systems resilience has emerged since the beginning of the COVID-19 pandemic, highlighting the disruptive nature that the pandemic has had on systems of care worldwide [41, 42]. In regards to mental health care systems specifically, research has shown that pandemic-related disruptions coincided with worsening symptoms, loss of usual systems of support, and poorer experiences with care [43, 44]. While our findings echo this existing research, the insights shared by participants nevertheless offer unique insight into health systems resilience in First Nations’ contexts. In particular, participants shared the importance of maintaining access to locally rooted, face-to-face programs that emphasize culture and identity. Such perspectives are important, as systems of care for mental health in First Nations communities are increasingly being defined by the principle of self-determination [45], and planning for future crises requires culturally-relevant data based in local perspectives to inform locally-led interventions. Thus, this research contributes to the larger discussion on health systems resilience by (i) validating the findings of the broader research within First Nations contexts, and (ii) extending the body of work by adding the unique perspectives of one First Nations community.

Within the community, the COVID-19 pandemic was associated with new challenges to the provision of mental wellness supports, including disruption to care arising from mandated stay-at-home requirements and social distancing measures. While remote care helped address gaps in access to mental wellness supports in the short term, echoing research with Indigenous communities worldwide [46], it was evident that some community members were being left behind in the “*care-from-home*” or virtual care revolution. In this study, several participants noted a decrease in the quality of care, with both clients and service providers finding it difficult to meaningfully connect over virtual platforms. Moreover, some participants expressed confusion about which services remained available and had difficulty navigating supports that were offered. Most striking, participants voiced concern that efforts to ensure mental wellness had been superseded by the acute concerns of the pandemic, leading to an inability to provide much needed supports, including those rooted in First Nations healing approaches, including community gatherings and ceremonies. Given the importance of providing culturally meaningful supports for many individuals experiencing mental distress and/or a substance use challenge [22], this represents a critical service gap. Taken together, these issues suggest that there has been a major disruption to service access for community members during the pandemic. Timely and barrier-free access to care are critical aspects to ensuring wellness for First Nations communities experiencing mental distress [5], the lapse of which may partially explain the increase in mental wellness and substance use challenges that were observed during the pandemic [20, 21, 32, 47].

Unfortunately, challenges to access may have been compounded by the impacts of pandemic related gathering restrictions on broader systems of support. For example, previous research has shown strong social connections to be an important enabling factor in the decision to seek support for First Nations individuals facing acute mental health challenges [48]. Thus, the disruption of strong social connections, which were identified by participants as a key community strength prior to the pandemic, removed an additional safety net that might be drawn from to ensure care for acute challenges was being accessed when needed.

Though we acknowledge that pandemic-related gathering restrictions were necessary to reduce COVID-19 transmission, it appears these initiatives had noticeable disruptive impacts on mental wellness promotion efforts. As such, public health responses to future crises of this nature should involve contingencies to ensure that those experiencing mental distress are still able to access required services with as little disruption as possible, and if preferred, in person. That said, a degree of flexibility in service delivery (i.e., remote services should remain available for those who prefer this option) would be beneficial given differing perceptions of risk that can exist during crises, as was noted by participants. For example, remote services may have several benefits, such as reducing stigma when seeking help by providing individuals with a confidential way to access supports [46], and by helping facilitate access to hard-to-reach services for those living in rural and remote locales, and thus may be suitable for some people. However, given the challenges that some people reported regarding virtual services, it may be important to develop ways to improve virtual care to ensure that both patients and service providers feel comfortable and engage in meaningful and beneficial interactions. One suggestion for ensuring strong virtual client-provider connections for individuals experiencing isolation is hybrid models, such as those where the initial visit occurs in person before transitioning to virtual settings [46, 49].

Importantly, our focus on emergent pandemic-related challenges should not downplay that the community had several notable successes during the pandemic. As discussed previously, several successful local initiatives were launched to support community members at the pandemic’s onset, including establishing a field hospital, delivery of food supports for those required to quarantine, and financial assistance to all residents in the case of employment disruptions. Initiatives such as these could form the basis for rapid responses during future crises. The pandemic period saw the implementation of successful initiatives that began before COVID-19, including the opening of a transition house for individuals in recovery for substance use challenges and the establishment of warming centres for individuals experiencing homelessness. These and other initiatives targeting at-risk community members demonstrate continued resourcefulness despite challenges to service provision arising from the pandemic.

Moreover, despite the challenges of the pandemic, participants noted the importance of deeply rooted community strengths that existed well before the pandemic. Participants emphasized relational strengths, where people support one another in times of need. Additionally, participants emphasized strengths within the system of services, including a strong network of local service providers that works together and responds rapidly to community crises and individual challenges. Locally nurtured capacities are a core component to ensuring mental wellness in ways consistent with the principles of self-determination and local governance [45, 50], and the existence of these strengths serve as evidence of the growing success of local systems of support that existed within the community before the pandemic. However, as shared by participants, the COVID-19 pandemic fundamentally altered the nature of these supports.

### Enhancing the resilience of local health systems for mental wellness

Participants made several suggestions for initiatives that could be adopted to ensure individuals are aware of and are able to connect with available services during times of uncertainty, including fan-out services, a centralized contact for coordinating fragmented supports within the community, and public awareness initiatives intended to reach a wide-ranging audience. While these examples explicitly deal with the disruption to mental wellness promotion caused by the pandemic, they may nevertheless be appropriate in other emergency scenarios that First Nations communities are increasingly at risk for, including natural disasters and severe weather (e.g., flooding; forest fires) [51]. As such, initiatives designed to reduce disruption to the provision of services are likely to be beneficial beyond the context of COVID-19 by ensuring the system of care is resilient to future crises.

Moreover, initiatives to promote mental wellness in times of uncertainty would benefit from building upon existing community strengths that endured the pandemic. Studies emphasize the important role for social supports in broadly supporting wellness for First Nations communities [52, 53]. In this research, participants noted that strong social bonds were essential to support mental wellness during the pandemic period by buffering the negative impacts of pandemic-related stressors. However, it was clear that participants’ experiences with social isolation varied; thus, programs offering supports to individuals vulnerable to social isolation during times of uncertainty (e.g., Elders; medically vulnerable individuals) would be helpful for ensuring that strong social connections endure during periods of social upheaval. Additionally, youth may benefit from specific youth-focused programming. As mentioned by youth group participants, programs incorporating cultural elements would provide opportunities for youth to continue to learn about culture and cultural practices from older generations, which is a key component of ensuring wellness during the transition to adulthood [54, 55].

Participants noted that those experiencing serious mental health challenges, including substance use challenges, were especially in need of supports, and research suggests that individuals experiencing substance use issues faced new challenges due to the pandemic, including heightened risk of overdose, riskier use patterns (e.g., using substance alone), and increased frequency of use [56]. During future crises, ensuring access to services such as detox centres and opioid replacement programs is essential for ensuring individuals experiencing substance use challenges can maintain their journey to wellness. Programs integrating culturally-based interventions for those experiencing serious mental health challenges would help ensure those experiencing these challenges can continue to connect with culture and identity to support wellness.

Finally, it is important to note that a focus on local context does not exclude a strong role for other levels of governance. Also required is a continuing commitment from policy makers within settler-oriented governments to honour the principles of reconciliation [57] by ensuring that First Nations have the resources necessary to provide for mental wellness promotion efforts in ways consistent with ideals of local governance and self-determination under all conditions [50]. This includes providing necessary support and resources for overstretched service providers during crises, including additional materials (i.e., protective equipment), staffing, and mental health supports for staff who are facing burnout.

### Ongoing initiatives in the community

Though this manuscript primarily reports on the influence of the COVID-19 pandemic on systems of care for mental health and substance use challenges, to provide more context to the reader, in this section we will discuss ongoing initiatives within the community. Members of the youth focus group, which began as a support group for community youth during the pandemic, have continued to provide research advice and oversight within the community. Additionally, several youth group members have become deeply involved in a range of research and knowledge sharing projects independent of the FNWI, offering their unique and valuable perspectives on youth wellness both within the community and in wider regional forums. Warming centres, which provided crucial support to community members experiencing housing insecurity during the pandemic, have continued to operate each winter. The FNWI continues to work closely with Saugeen First Nation on several community-oriented projects, including youth-focused participatory action research. Research evaluating the transition house for individuals exiting substance use treatment, which operated during the pandemic, is being planned.

### Strengths and limitations

A key strength of this study was the inclusion of several distinct yet complimentary viewpoints within the community, including people with lived experiences/those supporting loved ones with lived experiences, service providers, and local youth, which provided a broad and rich description of community-wide experiences with mental wellness during the COVID-19 pandemic. However, our study also has several limitations. Foremost, our analysis included few individuals who identified as men; thus, we may have excluded important experiences of men should they differ from those described in this article. Furthermore, data were gathered at different time points amid the COVID-19 pandemic; however, all data were integrated and interpreted congruently in the analysis. As such, it is possible that certain reported challenges and recommendations were more salient at some points of the pandemic than others and not representative of individuals’ COVID-19 experiences overall, given the evolution of pandemic-related restrictions and broader community experiences of COVID-19 over time. Another limitation is the small sample sizes of each data collection method; the two focus groups only contained five individuals each, which may impact the robustness of those findings. Moreover, only two individuals with lived experience were included in the one-to-one interviews, with the remaining seven participants being loved ones taking care of a person with lived experience. This may have made the perspectives of carers more prominent within the findings. Finally, as participants were directly recruited by the local coordinator/Knowledge Holder, it is possible that perspectives outside of the network of the coordinator were unintentionally excluded.

## Conclusion

The COVID-19 pandemic was associated with disruption to the provision of mental wellness supports within the First Nation community involved in this research. Though the acute phase of the COVID-19 pandemic has passed, the experiences shared by participants offer important lessons regarding the management of future crises. Ensuring that access to supports and services for mental wellness and/or substance use challenges can continue with as little disruption as possible should be a key factor for any mental wellness promotion efforts within First Nations communities. Additionally, the approach used to gather an understanding of mental wellness and substance use challenges demonstrates the importance of community-based research locally with First Nations communities, and provides opportunities for future adaptations within various community and organizational contexts. As suggested by participants, local initiatives to help individuals navigate services during crises are a valuable way to ensure access to care remains unimpeded.

## Supplementary Information

Additional File 1. Semi-structured interview guides.

Additional File 2. Focus group discussion guides.

## Data Availability

The data that support the findings of this study are not publicly available to maintain the confidentiality of research participants.

## Abbreviations

CAC: Community Advisory Circle
CAMH: Centre for Addiction and Mental Health
COVID-19: Coronavirus Disease– 2019
SFN: Saugeen First Nation
