# Supplementary material for: “I want to hear you talk with your heart”: perspectives on receiving and providing mental wellness supports during the COVID-19 pandemic within a First Nation community in Canada
Source: BMC Public Health. 2025 Aug 14;25:2780. doi: 10.1186/s12889-025-23723-y (PMC12351811; doi:10.1186/s12889-025-23723-y)
Supplement: Supplementary file 1 — Additional File 1. Semi-structured interview guides. [file 12889_2025_23723_MOESM1_ESM.pdf]

Interview Script for Interviews with PSLO

Study Title: First Nations Wellness Initiative: Mitigating the Impact of COVID-19

Turn on the tape recorder and begin the interview now.

Thank you for agreeing to participate in this project. The purpose of this interview is to obtain information about your loved one's experiences with services and supports for mental health and/or substance use and the impacts of the COVID-19 pandemic on their mental health and substance use issues and their experiences with services and supports. This could include formal treatment services or informal services and supports. We'd like to hear about their experiences receiving services as well as times when they've needed help but were unable to get it. Your input will be used to help us understand the strengths and weaknesses of the local system of care for mental health and substance use problems, and help us uncover ways that local services might be improved during the current pandemic and in future pandemics. Just to be clear -- we are doing these interviews for research purposes only, and my role in the interview is just to listen so that we can learn from people's experiences, it is not to judge you in any way or to provide feedback to you.

We are going to be recording the discussions so that we can review and summarize the things that are said today. I may also be taking some notes. Audio recordings will be transferred to a written document, and your name will be replaced by a pseudonym. All information discussed in this interview will remain confidential and will be used for research purposes only. Your identity will not be made known to the service providers involved in this research or other participants in this research. Your participation in this interview is voluntary. If any of the questions or topics covered in this discussion make you feel uncomfortable at any point in the interview, please let me know or feel free not to answer. I'd like to remind you that all the information that you provide us will be protected within the limits of the law, requiring mandatory reporting of child abuse.

STATE PARTICIPANT IDENTIFICATION NUMBER AT BEGINNING OF RECORDING

Participant ID: ____________________

GENDER of INTERVIEWEE:

GENDER OF THEIR LOVED ONE:

AGE OF INTERVIEWEE:

AGE OF THEIR LOVED ONE:

INTERVIEWEE'S RELATIONSHIP WITH LOVED ONE (e.g. partner, parent, child, sibling, friend, etc to loved one):

EXPERIENCES OF MENTAL HEALTH AND SUBSTANCE USE

1. You were invited to participate in this project because your loved one has experienced issues related to mental health and/or substance use. Can you explain the type of issue(s) they have experienced?

____
____
____
____

2. Have they been given a diagnosis from a health professional for their mental health and/or substance use?
3. Have they been given any other diagnoses relating to mental health and/or substance use?

EXPERIENCES GETTING HELP PRIOR TO THE PANDEMIC

MENTAL HEALTH ISSUES

4. At what age do you think your loved one first started to show signs or symptoms of having a mental health issue?

5. How did they become aware they had a mental health issue?

____
____
____
____

6. Who or where did your loved one first turn to for help? (Probe for formal and informal services)

____
____
____

7. Why did they choose to go to them over other people or places?

____
____
____

8. Who or where else did they turn to for help?

____
____
____

9. What about you, have you ever tried to get help for dealing with your loved one's mental health issue?

[If yes]

10. Who or where did you turn to for help? (Probe for formal and informal services)

____
____
____

11. Why did you choose to go to them over other people/places?

____
____
____

12. Did these people/places meet your needs as a family member?

____
____

SUBSTANCE USE ISSUES

13. Thinking now about your loved one's current problems or the problems they've had since being an adult, how long after they realized they had an issue with substance use was it before they tried to get help – either through formal services or other informal supports?

14. Who or where did they first turn to for help? (Probe for formal and informal services)
____
____
____

15. Why did they choose to go to them over other people or places?
____
____
____

16. Who or where else did they go to for help?
____
____
____

17. What about you, have you ever tried to get help for dealing with your family member's substance use?

[If yes]
18. Who or where did you turn to for help? (Probe for formal and informal services)
____
____
____

19. Why did you choose to go to them over other people/places?
____
____
____

20. Did these people/places meet your needs as a loved one?
____
____
____

NEGATIVE EXPERIENCES GETTING HELP

We will talk more about the specific services and supportive people your loved one went to for mental health or substance use in a few minutes, but first I want to get a sense of what your loved one's experiences trying to get help were like.

21. Thinking about times when your loved one really needed help, was there ever a time when they didn't get the help they wanted?

[If yes]
22. What prevented them from getting the help they wanted?
____
____
____
____

23. Thinking about the help they have received, have there been times when they did not have all their needs met?

[If yes]
24. What needs were not met?
____
____
____
____

25. What do you think might have been done to better meet their needs?
____
____
____
____

26. Was there anything that happened while they were getting help that made their issues worse?

[If yes]

27. What happened?
____
____
____
____

28. Has your loved one ever achieved recovery/overcome their mental health/substance use issues, if even for a short period of time?

[If yes]
29. Did they ever relapse/did their mental health/substance use issues return?

[If yes]

30. What do you think contributed to their relapse/why did the mental health/substance use issues return?

____
____
____
____

31. In general, what do you think are the main barriers/challenges that your loved one faced in trying to receive help for their issues?

____
____
____
____

POSITIVE EXPERIENCES GETTING HELP

32. Was there anything that your loved one found especially helpful or that made getting help easier or better for them?

[If yes]

33. What made it easier?

____
____
____
____

34. Was there anyone or any program that was especially helpful to your loved one?

[If yes]

35. What was helpful?

____
____
____
____

36. What were the best experiences they had while getting help for mental health or substance use?

____
____
____
____

37. Thinking about the places where your loved one has received help, which places would they most likely go again?

____
____
____
____

[For informal supports, if applicable]

38. Who would they most likely talk to again?

____
____
____
____

[If achieved recovery]

39. You mentioned previously that your loved one achieved recovery/was able to overcome their mental health/substance use issues. How did they manage to sustain it as long as they did?

____
____
____
____

IMPACTS OF PANDEMIC

Now I'd like talk to you about how the COVID-19 pandemic may have affected you during the last several months.

40. Does your loved one have concerns about COVID-19?

- Probe: If yes: What are their concerns?
- If no: Why? (ask them to elaborate on why their loved one doesn't have concerns of COVID-19)

____
____
____
____

41. Were they able to self-isolate during COVID?
    - Probe: If yes: Under what circumstances did they choose to self-isolate? What did they do to self-isolate? How has this impacted them? Has their substance use

become riskier? (e.g., Using alone? Overdoses? Criminalization? Wash their hands as needed?)

- If no: how come? Ask them to elaborate on why their loved one hasn't self-isolated. (E.g., unable to? Don't understand/agree with the importance? Employment not permitting?) Have they experienced any issues with the law?)

____
____
____
____

42. Has COVID-19 impacted your loved one's ability to make money? Has it impacted their social groups in any way?
    - Probe: If yes: In which ways has it impacted them? (e.g., food security? loss of job/income? increased risky behaviors to gain income? Unable to utilize/rely on social networks?) What, if anything, have they done to cope/deal with this? Do they have protective gear?
    - If no: How come? Ask them to elaborate on how their loved one's social and financial well-being has remained the same.

____
____
____
____

43. Given how your loved one self-identifies, do you feel like they are impacted in specific ways, in comparison to others?
    - Probe: If yes: How so? How do they identify? (e.g. if they say as a PWLE, ask them if there are other ways that they also identify)
    - Probe: If no: How come? How do they identify?

____
____
____
____

44. Has COVID-19 impacted your loved one's physical, emotional, spiritual or mental health?
    - Probe: If yes: In which ways has it impacted them? Has there been anything specific that has impacted an existing mental health issue? (e.g., Is their drug use putting them at risk? Other physical issues? Increased stress? Feelings of safety?). What, if anything, have they done to cope/deal with this?

- Has this impact on physical or mental health affected their ability to access medication for OAT and/or psychiatric medications? If so, in which ways?
- Are they able to have access to cultural supports? (e.g. elders, knowledge translators, sacred medicines, smudge?)
- If no: How come? Ask them to elaborate on how their loved one's physical and/or mental health has remained the same.

____
____
____
____

45. Has COVID-19 changed your loved one's substance use?
    - Probe: If yes: in which ways? (Increased? Decreased? Types of substance? Route of administration? Location of use?). Has this effect been negative? If so, what, if anything, have they done to cope/deal with this?
    - If no: what does their substance use look like? (Types of substance? Route of administration? Location of use?). Ask them to elaborate on why and how it has stayed the same.
    - Probe: Are they now more or less likely to use alone? Have they been using with other people in other ways, for example with other people over the phone? Do you feel like they are more or less at risk for overdose right now?
    - Probe: Did they get their drugs on reserve or elsewhere before the pandemic? Has that changed? Has it changed in terms of Substance? Demand? Cost?)
      What, if anything, have they done to cope/deal with this? Are they aware of what safe supply is?
    - If no change: why? (ask them to elaborate on why and how it has stayed the same)

____
____
____

46. What services have they been able to access during the pandemic? What services have they not been able access during the pandemic? How has this affected them? How has this affected you?

____
____
____
____

47. A) Have they been able to access any of the **substance use** related services they would normally access since COVID-19? (e.g., OAT, supervised consumption sites, needle

exchange, addiction clinics, drop-in groups, etc.)

- Probe: If yes: Which services are you referring to? Have any of these services changed at all since COVD-19? If so, how have they changed? Ask them to elaborate on if and which ways the services their loved one's use have changed (e.g., hours, locations, personnel, etc.)
- Have they had to do things they wouldn't normally do, like re-use needles or syringes?
- Have the increased barriers impacted their treatment (i.e. not being able to access services as often as desired, difficulties traveling to services, etc.)
- If no: Can you please describe why they haven't been able to access services and how this has affected them? How has it affected you? Ask them to elaborate on any problems their loved one may have faced accessing services. What, if anything, have they done to cope/deal with this?

____
____
____
____

48. Have they been able to access **any other services** they would normally access since COVID-19? (e.g., health services, government services, employment services, doctor's appointments, pharmacies, ID services etc.)

- Probe: If yes: Which services are you referring to? Have any of these services changed at all since COVD-19? Ask them to elaborate on if and which ways the services their loved one uses have changed (e.g., hours, locations, personnel, etc.)
- Have they sought care for COVID symptoms? If so, how did they seek care? Were they tested? Were they provided supports for self-isolation? Etc.
- If no: Can you please describe why they haven't been able to access services and how this has affected them? How has this affected you? Ask them to elaborate on any problems their loved one may have faced accessing services. What, if anything, have they done to cope/deal with this?

____
____
____
____

49. What helped your loved one to cope with these combined challenges of the pandemic as well as MHS challenges?

____
____

____
____

50. What helped you to cope with these combined challenges of the pandemic as well as your loved one's MHS challenges?

____
____
____
____

51. What changes have you noticed in your loved one during the pandemic? Yourself? The community?

____
____
____
____

52. What kinds of supports has SFN been able to provide to the community? How have these helped? What could have been done better?

____
____
____
____

53. What kinds of supports does SFN need in the community at this time of crisis and in the long-term?

____
____
____
____

54. Did things become easier for your loved one when the lockdowns were lifted and people were able to return to work and children were able to go back to school? How did this affect your loved one's MHS issues? Did things become easier for you?

____
____
____
____

55. In light of everything you mentioned, can you suggest some things that would be helpful for your loved one during the current COVID-19 pandemic? (e.g., Related to substance use? Supply? Service utilization and access? Regarding self-isolation? Socially/economically? Physically/mentally?)

- Probe: Do you think a prescribed, safe supply of substances would be helpful now?

56. Is there anything else that you want to discuss related to COVID-19 and how it has impacted your loved one's substance use, ability to access services, or overall well-being?
    - Probe: Is there anything that we are not asking, that you would have liked us to ask?

57. Based on your loved one's and your own experiences, what suggestions do you have for ways to improve services and supports for people in [community] who have mental health, and/or substance use problems during the pandemic? (Probe for access, treatment programs, service delivery, referral systems, communication between programs, service integration, etc)

Interview Script for Interviews with PWLE

Study Title: First Nations Wellness Initiative: Mitigating the Impact of COVID-19

Turn on the tape recorder and begin the interview now.

Thank you for agreeing to participate in this project. The purpose of this interview is to obtain information about your experiences with services and supports for mental health and/or substance use and the impacts of the COVID-19 pandemic on your mental health and substance use issues and your experiences with services and supports. This could include formal treatment services or informal services and supports. We'd like to hear about your experiences receiving services as well as times when you've needed help but were unable to get it. Your input will be used to help us understand the strengths and weaknesses of the local system of care for mental health and substance use problems and help us uncover ways that local services might be improved during the current pandemic and in future pandemics. Just to be clear -- we are doing these interviews for research purposes only, and my role in the interview is just to listen so that we can learn from people's experiences, it is not to judge you in any way or to provide feedback to you.

We are going to be recording the discussions so that we can review and summarize the things that are said today. I may also be taking some notes. Audio recordings will be transferred to a written document, and your name will be replaced by a pseudonym. All information discussed in this interview will remain confidential and will be used for research purposes only. Your identity will not be made known to the service providers involved in this research or other participants in this research. Your participation in this interview is voluntary. If any of the questions or topics covered in this discussion make you feel uncomfortable at any point in the interview, please let me know or feel free not to answer. I'd like to remind you that all the information that you provide us will be protected within the limits of the law, requiring mandatory reporting of child abuse.

STATE PARTICIPANT IDENTIFICATION NUMBER AT BEGINNING OF RECORDING

Participant ID: ____________________

GENDER:

AGE:

EXPERIENCES OF MENTAL HEALTH AND SUBSTANCE USE

1. You were invited to participate in this project because you have experienced issues related to mental health and/or substance use. Can you explain the type of issue(s) you've experienced?

____
____
____
____

2. Have you been given a diagnosis from a health professional for your mental health and/or substance use?

3. Have you been given any other diagnoses relating to mental health and/or substance use?

EXPERIENCES GETTING HELP PRIOR TO THE PANDEMIC

MENTAL HEALTH ISSUES

4. At what age did you first realize you had a mental health issue?

5. How did you become aware you had a mental health issue?

____
____
____
____

6. Who or where did you first turn to for help? (Probe for formal and informal services)

____
____
____
____

7. Why did you choose to go to them over other people or places?

____
____

____
____

8. Who or where else did you turn to for help?

____
____
____
____

SUBSTANCE USE ISSUES

9. Thinking now about your current problems or the problems you've had since being an adult, how long after you realized you had an issue with substance use was it before you tried to get help – either through formal services or other informal supports?

10. Who or where did you first turn to for help? (Probe for formal and informal services)

____
____
____
____

11. Why did you choose to go to them over other people or places?

____
____
____
____

12. Who or where else did you go to for help?

____
____
____
____

NEGATIVE EXPERIENCES GETTING HELP

We will talk more about the specific services and supportive people you went to for mental health or substance use in a few minutes, but first I want to get a sense of what your experiences trying to get help were like.

13. Thinking about times when you really needed help, was there ever a time when you didn't get the help you wanted?

[If yes]
14. What prevented you from getting the help you wanted?
____
____
____
____

15. Thinking about the help you have received, have there been times when you felt that you did not have all your needs met?

[If yes]
16. What needs were not met?
____
____
____
____

17. What do you think might have been done to better meet your needs?
____
____
____
____

18. Was there anything that happened while you were getting help that made your issues worse?

[If yes]
19. What happened?
____
____
____
____

20. Have you ever achieved recovery/overcome your mental health/substance use issues, if even for a short period of time?

[If yes]
21. Did you ever relapse/did your mental health/substance use issues return?

[If yes]
22. What do you think contributed to the relapse/why did the mental health/substance use issues return?
____
____

____
____

23. In general, what do you think are the main barriers/challenges that you faced in trying to receive help for your issues?

____
____
____
____

POSITIVE EXPERIENCES GETTING HELP

24. Was there anything that you found especially helpful or that made getting help easier or better for you?

[If yes]
25. What made it easier?

____
____
____
____

26. Was there anyone or any program that was especially helpful to you?

[If yes]
27. What was helpful?

____
____
____
____

28. What were the best experiences you had while getting help for mental health or substance use?

____
____
____
____

29. Thinking about the places where you have received help, which places would you most likely go again?

____
____
____
____

[For informal supports, if applicable]

30. Who would you most likely talk to again?

____
____
____
____

[If achieved recovery]

31. You mentioned previously that you achieved recovery/were able to overcome your mental health/substance use issues. How did you manage to sustain it as long as you did?

____
____
____
____

IMPACTS OF PANDEMIC

Now I'd like talk to you about how the COVID-19 pandemic may have affected you during the last several months.

32. Do you have concerns about COVID-19?
    - Probe: If yes: What are your concerns?
    - If no: Why? (ask them to elaborate on why they don't have concerns of COVID-19)

____
____
____
____

33. Were you able to self-isolate during COVID?
    - Probe: If yes: Under what circumstances did you choose to self-isolate? What did you do to self-isolate? How has this impacted you? Has your use become riskier? (e.g., Using alone? Overdoses? Criminalization? Wash your hands as needed?)
    - If no: how come? Ask them to elaborate on why they haven't self-isolated. (E.g., unable to? Don't understand/agree with the importance? Employment not permitting?) Have you experienced any issues with the law?)

____
____
____
____

34. Has COVID-19 impacted your ability to make money? Has it impacted your social groups in any way?

- Probe: If yes: In which ways has it impacted you? (e.g., food security? loss of job/income? increased risky behaviors to gain income? Unable to utilize/rely on social networks?) What, if anything, have you done to cope/deal with this? Do you have protective gear?
- If no: How come? Ask them to elaborate on how their social and financial well-being has remained the same.

____
____
____
____

35. Given how you self-identify, do you feel like you are impacted in specific ways, in comparison to others?
    - Probe: If yes: How so? How do you identify? (e.g. if they say as a PWLE, ask them if there are other ways that they also identify)
    - Probe: If no: How come? How do you identify?

____
____
____
____

36. Has COVID-19 impacted your physical, emotional, spiritual or mental health?
    - Probe: If yes: In which ways has it impacted you? Has there been anything specific that has impacted an existing mental health issue? (e.g., Is your drug use putting you at risk? Other physical issues? Increased stress? Feelings of safety?). What, if anything, have you done to cope/deal with this?
    - Has this impact on physical or mental health affected your ability to access medication for OAT and/or psychiatric medications? If so, in which ways?
    - Are you able to have access to cultural supports? (e.g. elders, knowledge translators, sacred medicines, smudge?)
    - If no: How come? Ask them to elaborate on how their physical and/or mental health has remained the same

____
____
____
____

37. Has COVID-19 changed your substance use?

- Probe: If yes: in which ways? (Increased? Decreased? Types of substance? Route of administration? Location of use?). Has this effect been negative? If so, what, if anything, have you done to cope/deal with this?
- If no: what does your substance use look like? (Types of substance? Route of administration? Location of use?). Ask them to elaborate on why and how it has stayed the same.
- Probe: Are you now more or less likely to use alone? Have you been using with other people in other ways, for example with other people over the phone? Do you feel like you are more or less at risk for overdose right now?
- Probe: Did you get your drugs on reserve or elsewhere before the pandemic? Has that changed? Has it changed in terms of Substance? Demand? Cost?)
- What, if anything, have you done to cope/deal with this? Are you aware of what safe supply is?
- If no change: why? (ask them to elaborate on why and how it has stayed the same)

____
____
____
____

38. What services have you been able to access during the pandemic? What services have you not been able to access during the pandemic? How has this affected you?

____
____
____
____

39. Have you been able to access any of the **substance use** related services you would normally access since COVID-19? (e.g., OAT, supervised consumption sites, needle exchange, addiction clinics, drop-in groups, etc.)
    - Probe: If yes: Which services are you referring to? Have any of these services changed at all since COVD-19? If so, how have they changed? Ask them to elaborate on if and which ways the services they use have changed (e.g., hours, locations, personnel, etc.)
    - Have you had to do things you wouldn't normally do, like re-use needles or syringes?
    - Have the increased barriers impacted your treatment (i.e. not being able to access services as often as desired, difficulties traveling to services, etc.)
    - If no: Can you please describe why you haven't been able to access services and how this has affected you? Ask them to elaborate on any problems they may have faced accessing services. What, if anything, have you done to cope/deal with this?

____
____
____
____

40. Have you been able to access **any other services** you would normally access since COVID-19? (e.g., health services, government services, employment services, doctor's appointments, pharmacies, ID services etc.)
    - Probe: If yes: Which services are you referring to? Have any of these services changed at all since COVD-19? Ask them to elaborate on if and which ways the services they use have changed (e.g., hours, locations, personnel, etc.)
    - Have you sought care for COVID symptoms? If so, how did you seek care? Were you tested? Were you provided supports for self-isolation? Etc.
    - If no: Can you please describe why you haven't been able to access services and how this has affected you? Ask them to elaborate on any problems they may have faced accessing services. What, if anything, have you done to cope/deal with this?

____
____
____
____

41. What helped you to cope with these combined challenges of the pandemic as well as MHS challenges?

____
____
____
____

42. What changes have you noticed in yourself during the pandemic? Your family (nuclear, extended, surrogate)? The community?

____
____
____
____

43. What kinds of supports has SFN been able to provide to the community? How have these helped? What could have been done better?

____
____
____
____

44. What kinds of supports does SFN need in the community at this time of crisis and in the long-term?

____
____
____
____

45. Did things become easier when the lockdowns were lifted and people were able to return to work and children were able to go back to school? How did this affect your MHS issues?

____
____
____
____

46. In light of everything you mentioned, can you suggest some things that would be helpful for you during the current COVID-19 pandemic? (e.g., Related to substance use? Supply? Service utilization and access? Regarding self-isolation? Socially/economically? Physically/mentally?)
    - Probe: Do you think a prescribed, safe supply of substances would be helpful now?

____
____
____
____

47. Is there anything else that you want to discuss related to COVID-19 and how it has impacted substance use, ability to access services, or overall well-being?
    - Probe: Is there anything that we are not asking, that you would have liked us to ask?

____
____
____
____

48. Based on your own experiences, what suggestions do you have for ways to improve services and supports for people in [community] who have mental health and/or substance use problems during the pandemic? (Probe for access, treatment programs, service delivery, referral systems, communication between programs, service integration, etc)

____
____
____
____
