# Supplementary material for: “I want to hear you talk with your heart”: perspectives on receiving and providing mental wellness supports during the COVID-19 pandemic within a First Nation community in Canada
Source: BMC Public Health. 2025 Aug 14;25:2780. doi: 10.1186/s12889-025-23723-y (PMC12351811; doi:10.1186/s12889-025-23723-y)
Supplement: Supplementary file 2 — Additional File 2. Focus group discussion guides. [file 12889_2025_23723_MOESM2_ESM.pdf]

Script for Service Provider Discussion Groups

Study Title: First Nations Wellness Initiative: Mitigating the Impact of COVID-19

Introduction

You were asked to participate in this discussion because of your role in the local system of care for mental health and/or substance use (MHS) challenges for members of the community. This discussion session is part of a larger project that aims to collect local data to inform the development of a wellness strategy in this community, with a specific focus on addressing the impacts of the COVID-19 pandemic. We are also conducting interviews with people with lived experience (PWLE) with MHS challenges as well as people who have loved ones with MHS challenges (PSLO). This part of the project examines the perspective of service providers.

The goal of this discussion is to gain a better understanding of how the system of services is functioning for members of your community during the current pandemic and identify ways this system can be improved in the current and future pandemics. Your participation will help us understand how the pandemic has affected the local network of services for MHS problems and will allow us to identify strengths as well as gaps and barriers in the overall system of care. This discussion group format will also allow participants to share information with one another and consider, as a group, ways the system might function better. [Sign consent forms]

The main focus of this discussion is on how the system of care is helping people from the community during the pandemic. So the questions pertain to band members who may or may not live on the reserve. Also, when we speak of MHS problems, we are referring to both single disorders as well as co-occurring disorders.

We'd like to start by having each participant introduce her or himself and tell us a little bit about their service or program and their role in the service/program.

STRENGTHS AND CHALLENGES PRIOR TO COVID-19 PANDEMIC

1. First, let's talk about the strengths of the system of care for people with problems related to mental health and substance use/addiction that existed prior to the pandemic. What do you see as the major strengths of the system and sources of resilience for people from the community?

2. What were the challenges and barriers that people from the community faced prior to the pandemic when getting help for MHS problems? What were the challenges that service providers faced in trying to provide appropriate services to the community?

IMPACTS OF THE PANDEMIC

3. Do you have concerns about COVID-19?
    - Probe: If yes: What are your concerns?

- If no: Why? (ask them to elaborate on why they don't have concerns of COVID-19)

4. Were community members that you serve able to self-isolate during COVID?

- Probe: If yes: Under what circumstances did they choose to self-isolate? What did they do to self-isolate? How has this impacted them? Has use become riskier? (e.g., Using alone? Overdoses? Criminalization? Wash hands as needed?)
- If no: how come? Ask them to elaborate on why they haven't self-isolated. (E.g., unable to? Don't understand/agree with the importance? Employment not permitting?) Have they experienced any issues with the law?)

5. Has COVID-19 impacted the community's physical, emotional, spiritual or mental health?
   - Probe: If yes: In which ways has it impacted the community? Has there been anything specific that has impacted an existing mental health issue? (e.g., Is drug use putting people at risk? Other physical issues? Increased stress? Feelings of safety?). What, if anything, have community members done to cope/deal with this?
   - Has this impact on physical or mental health affected ability to access medication for OAT and/or psychiatric medications? If so, in which ways?
   - Are community members able to have access to cultural supports? (e.g. elders, knowledge translators, sacred medicines, smudge?)
   - If no: How come? Ask them to elaborate on how physical and/or mental health has remained the same

6. Has COVID-19 changed substance use within the community?
   - Probe: If yes: in which ways? (Increased? Decreased? Types of substance? Route of administration? Location of use?). Has this effect been negative? If so, what, if anything, has been done to done to cope/deal with this?
   - If no: what does substance use within the community look like? (Types of substance? Route of administration? Location of use?). Ask them to elaborate on why and how it has stayed the same.
   - Probe: Are community members now more or less likely to use alone? Have they been using with other people in other ways, for example with other people over the phone? Do you feel like they are more or less at risk for overdose right now?
   - Probe: Did people get their drugs on reserve or elsewhere before the pandemic? Has that changed? Has it changed in terms of Substance? Demand? Cost?) What, if anything, have they done to cope/deal with this? Are they aware of what safe supply is?
   - If no change: why? (ask them to elaborate on why and how it has stayed the same)

7. Thinking about the services you provide to people who have MHS problems, what challenges are you facing that are unique to the pandemic?

8. What services have you been able to provide to the community? How have these helped? What could have been done better?

9. What is being done differently? What services are not being provided because of the pandemic? How has this affected the MHS issues in the community?

10. What kinds of supports has SFN been able to provide to the community? How have these helped? What could have been done better?

11. Have people seeking help been able to access any of the **substance use** related services they would normally access since COVID-19? (e.g., OAT, supervised consumption sites, needle exchange, addiction clinics, drop-in groups, etc.)
    - Probe: If yes: Which services are you referring to? Have any of these services changed at all since COVD-19? If so, how have they changed? Ask them to elaborate on if and which ways the services have changed (e.g., hours, locations, personnel, etc.)
    - Have community members using drugs had to do things you wouldn't normally do, like re-use needles or syringes?
    - Have the increased barriers impacted treatment (i.e. not being able to access services as often as desired, difficulties traveling to services, etc.)?
    - If no: Can you please describe why community members haven't been able to access services and how this has affected them? Ask them to elaborate on any problems community members may have faced accessing services. What, if anything, have community members done to cope/deal with this?

12. Have community members been able to access **any other services** they would normally access since COVID-19? (e.g., health services, government services, employment services, doctor's appointments, pharmacies, ID services etc.)
    - Probe: If yes: Which services are you referring to? Have any of these services changed at all since COVD-19? Ask them to elaborate on if and which ways the services community members use have changed (e.g., hours, locations, personnel, etc.)
    - Have community members been able to seek care for COVID symptoms? If so, how do they seek care? Are they tested? Were they provided supports for self-isolation? Etc.

      If no: Can you please describe why community members haven't been able to access services and how this has affected them? Ask them to elaborate on any problems community members may have faced accessing services. What, if anything, have they done to cope/deal with this?

13. What kinds of supports does SFN need in the community at this time of crisis and in the long-term?

14. How have you and your colleagues been affected by the pandemic (e.g., burnout)? What supports are needed for service providers to help them to address these challenges?

15. Do you have a self-care plan? Please describe your self-care plan.

16. In light of everything you mentioned, can you suggest some things that would be helpful for PWLE during the current COVID-19 pandemic? (e.g., Related to substance use? Supply? Service utilization and access? Regarding self-isolation? Socially/economically? Physically/mentally?)
    - Probe: Do you think a prescribed, safe supply of substances would be helpful now?

17. Is there anything else that you want to discuss related to COVID-19 and how it has impacted mental health and/or substance use, ability to access services, or overall well-being?
18. Probe: Is there anything that we are not asking, that you would have liked us to ask?

OVERALL SUGGESTIONS/RECOMMENDATIONS

19. What can be done during and after the pandemic to improve the services and the overall system for people from the community who have MHS problems?

20. Have there been any changes due to the pandemic that are likely to have an impact on services for people who have MHS problems?

21. Are there any lessons learned from the pandemic that should be considered if we face another pandemic like this?

First Nations Wellness Initiative - Community Focus Group Questions

Introduction

Thank you for agreeing to participate in this focus group. You were asked to participate in this discussion because of your experiences related to mental health and/or substance use (MHS) challenges for members of the community. This discussion session is part of a larger project that aims to collect local data to inform the development of a wellness strategy in this community, with a specific focus on addressing the impacts of the COVID-19 pandemic.

The goal of this discussion is to help identify what is most needed in the community to address substance use and/or mental health challenges in the community as well as negative effects of the pandemic, and identify strategies that might be used to promote wellness using existing sources of strength. This discussion group format will also allow participants to share information with one another and consider, as a group, ways the system might function better.

We are going to be recording the discussion so that we can review and summarize the things that are said today. I may also be taking some notes. You should not mention names or reveal the identify of any individuals during the discussion. This recording will be transferred to a written document, and your names and the names of other individuals will not be used. All information discussed in this interview will remain confidential and will be used for research purposes only. Your participation in this discussion is voluntary. If any of the questions or topics covered in this discussion make you feel uncomfortable at any point in the discussion, please let me know or feel free not to answer.

Please do not disclose information about other participants or the contents of the discussion with other people after the discussion group.

Are there any questions?

Do you still wish to continue with the discussion? (yes/no). [If anyone says no] Thank you. If you no longer wish to participate, we cannot continue the discussion. We will wait for you to leave the room before we begin.

Are you okay with the interview being audio taped (yes/no).

[If anyone says no] Thank you, but we cannot continue the discussion without audio recording. We will wait for you to leave the room before we begin.

[If all say yes] Thank you, now I will start the recording and begin the discussion…

1. What issues affect people's mental health and substance use in [community]? How has the COVID-19 pandemic affected mental health and substance use for people in [community]? How has the pandemic affected services provided to people who experience mental health and substance use challenges?

2. What do you think is needed to improve mental health and substance use issues? What would help diverse people (i.e., people of different ages and genders) in [community]?

3. What are strengths that [community] can draw on to address mental health and substance use challenges?
